# Essential Thrombocythemia Possible Cause of Ischemic Cerebrovascular Disease: A Case Report

**DOI:** 10.7759/cureus.71846

**Published:** 2024-10-19

**Authors:** Ibrahim Korucu, Muhammed Fatih Ciril

**Affiliations:** 1 Department of Neurology, Mardin Training and Research Hospital, Mardin, TUR; 2 Department of Emergency Medicine, Mardin Training and Research Hospital, Mardin, TUR

**Keywords:** : cerebrovascular disease, chronic myeloproliferative neoplasms, essential thrombocythaemia, jak2 v617f mutation, vascular neurology

## Abstract

Essential thrombocythemia (ET) is a rare, chronic myeloproliferative neoplasm characterized by the overproduction of platelets. ET is of significant clinical importance due to thrombotic and hemorrhagic cerebrovascular disease. The JAK2 V617F mutation has been identified in approximately 50-60% of ET cases. Our case report: a 41-year-old male presented to the hospital with a one-day history of vertigo, ataxic gait, and vomiting. The patient was diagnosed with ET at an external center two years ago. The JAK2 V617F mutation was detected. He regularly uses acetylsalicylic acid 100 mg per day. Magnetic resonance imaging (MRI) showed an acute infarction involving the bilateral cerebellar hemisphere, thalamic area, and right occipital area. Computed Tomography Angiography showed that no significant stenosis was detected in major branches. The patient was diagnosed with ischemic cerebrovascular disease for ET. Antiplatelet therapy was started with acetylsalicylic acid 100 mg and clopidogrel 75 mg once a day. With the recommendation of hematology, cytoreductive treatment, hydroxyurea 1000 mg twice a day, was started. The patient's complaints were resolved at the end of the second day, and the patient with minimal ataxia was discharged with recommendations. Patients with ET should be aware of ischemic cerebrovascular disease and consider antiplatelet and cytoreductive treatment options.

## Introduction

Essential thrombocythemia (ET) is a rare, chronic myeloproliferative neoplasm characterized by excessive production of platelets [1]. In 2005, it was reported that a somatic gain-of-function mutation in the Janus kinase (JAK2) gene, resulting in the replacement of valine by phenylalanine at codon 617 (V617F), contributes to the clonal expansion of hematopoietic cells in myeloproliferative neoplasms. The JAK2 V617F mutation is found in approximately 50-60% of patients with essential thrombocythemia. [2]. Although most ET patients are asymptomatic, the risk of developing thrombosis is more than 20% [3]. 80-90% of thrombotic supplements are arterial, rarely venous. [4]. ET has significant clinical importance due to thrombotic and hemorrhagic cerebrovascular disease [5]. In this article, we aimed to explain ET-cerebrovascular disease through an ET patient whom we kindly referred to the emergency department.

## Case presentation

**Figure 1:**
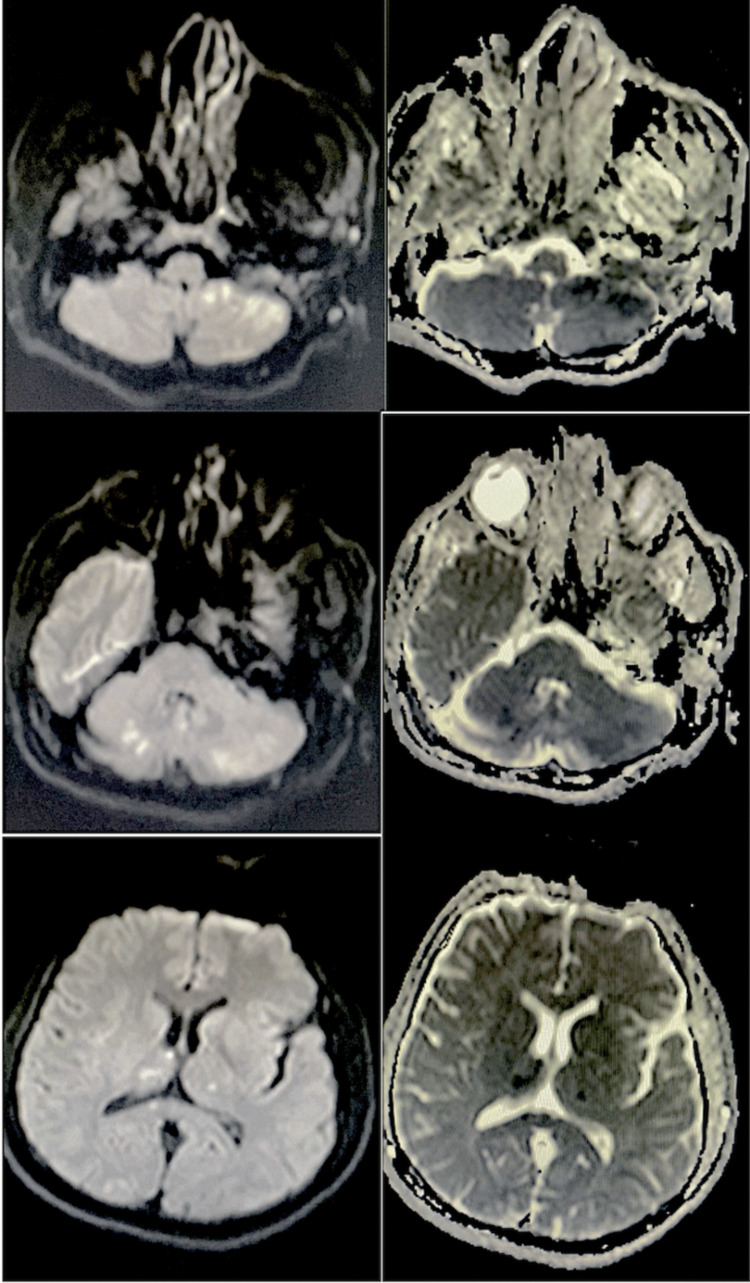
Magnetic resonance imaging (MRI) showed an acute infarction involving the bilateral cerebellar hemisphere and corpus callosum, thalamic area, and right occipital area

**Figure 2:**
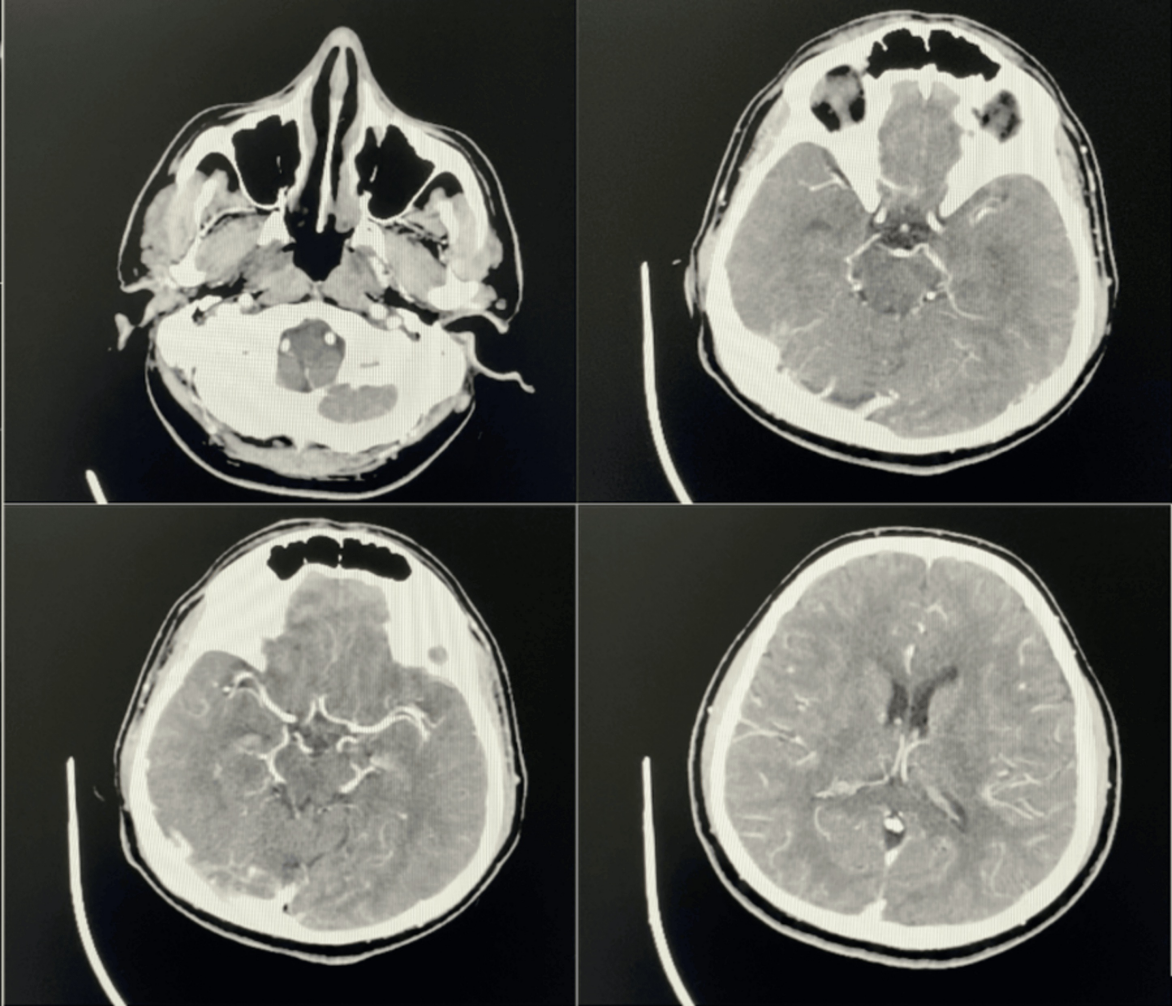
Computed tomography angiography showed that no significant stenosis was detected in major branches

A 41-year-old man was admitted to the hospital with a history of vertigo, ataxic gait, and vomiting that had been going on for a day. The patient was diagnosed with ET two years ago at an external center. The JAK2 V617F mutation was detected. Both of his siblings have the same diagnosis. There are no other known comorbidities. He regularly uses 100 mg of acetylsalicylic acid (ASA) per day. The patient's systemic examination was normal. On neurological examination, evoked horizontal nystagmus and ataxic gait were present. No other pathological findings on neurological examination. The patient's NIHSS score was 2 and mRS 1. Complete blood count was abnormal; platelet count was 849,000/μl, total leukocyte count was 12,670/μl, and hemoglobin was 14.6 g/dL. Magnetic Resonance Imaging (MRI) showed acute infarction involving bilateral cerebellar hemispheres and thalamic area, right occipital area (Figure 1). The patient was admitted to the neurology service with the diagnosis of ischemic cerebrovascular disease. Due to the lateness of his application, he was not a candidate for any revascularization procedure. No thrombus was detected on transthoracic echocardiography and no other pathologic findings were detected. No significant stenosis was detected in the major branches of computed tomography angiography (Figure 2). No additional imaging was performed aside from these scans. In the 48-hour rhythm hall, the basal rhythm was sinus. No arrhythmias were detected. The patient was taking 100 mg of aspirin daily, and in addition, 75 mg of clopidogrel was added for antiplatelet therapy once a day. Upon the recommendation of the hematology department, 1000 mg hydroxyurea twice a day was started as cytoreductive treatment. At the end of the second day, the patient's complaints had diminished. The patient, with an NIHSS score of 1 and an mRS score of 0, exhibited minimal ataxia. Neurology and hematology outpatient follow-up was recommended for the patient.

## Discussion

There are studies in the literature that demonstrate microcirculatory pathology, the role of high leukocyte and red blood cell counts, and the impact of the JAK2V617F mutation in the etiology of neurological symptoms associated with ET. The hypothesis of reversible platelet aggregation due to signaling via the arachidonic acid cascade and cyclooxygenase-1 (COX-1) activation may be consistent with the clinical benefit of antithrombotic agents. In ischemic stroke, antiplatelet therapy is generally recommended within the first 24-48 hours. If the platelet count before treatment is >1,000,000-1,500,000/mm3, cases of acquired von Willebrand disease with ET should be excluded before starting ASA. [6,7]. There are few indications of dual antiplatelet therapy (DAPT) in ischemic stroke and transient ischemic attack (TIA). DAPT is generally recommended for small noncardioembolic ischemic strokes (NIHSS score ≤ 3). It is also recommended to continue treatment for three weeks for TIAs not receiving thrombolytic therapy and for three months for ischemic stroke or TIA due to stenosis of a large intracranial artery (70-99%). After this period, lifelong use of a single antiplatelet agent is recommended for secondary stroke prevention. [8,9]. For cardiovascular (CV) protection in ET, low-dose ASA twice daily is recommended in arterial thrombotic diseases if there are additional risk factors, such as age >60 years or the presence of JAK 2 mutation or CV risk factors [10]. ET patients presenting with non-cardioembolic stroke/TIA with any of the risk factors mentioned above should receive DAPT for three weeks to three months, and low-dose ASA twice daily is recommended to reduce recurrent ischemic strokes. Oral anticoagulant therapy is recommended for the prevention of secondary stroke within 14 days from the onset of cardioembolic stroke due to atrial fibrillation. Our case has a definitive diagnosis of essential thrombocythemia (ET) based on the presence of a gene mutation. Given that the necessary investigations for cardiovascular and vascular pathologies returned negative results, an ischemic event primarily attributed to ET was considered. However, these cases should be thoroughly examined from a cardiological standpoint, as the literature includes cardioembolic cases that also affect similar areas of imaging studies [11].

In addition to antiaggregant therapy, cytoreductive therapy is indicated in high-risk categories to prevent thrombotic complications. The usual treatment goal for cytoreductive therapies is a platelet count between 100,000/mm3 and 400,000/mm3 [12]. Hydroxyurea (HU) is preferred as it has been proven to reduce the risk of thrombosis in randomized control trials in high-risk patients comparing HU versus no myelosuppressive therapy [13,14]. Due to its gonadal toxicity, mutagenicity, and teratogenic effects, HU is generally avoided in young individuals, women of childbearing age, and pregnant patients. Noninferiority of anagrelide compared with HU has been established; however, it has been associated with side effects such as myelofibrosis and cardiac events [15]. Busulfan, interferon, and ruxolitinib are also used as alternative cytoreductive therapies [11,16].

## Conclusions

Patients presenting with ischemic stroke should have their history thoroughly investigated, and potential underlying causes should be carefully considered. In cases like ours, it is important to remember that patients with a diagnosis of essential thrombocythemia (ET) have a predisposition to thrombosis. Even in patients who are on prophylactic aspirin, such thrombotic events can occur, necessitating further imaging studies. Additionally, consideration should be given to transitioning to supplementary therapies, such as cytoreductive medications (e.g., hydroxyurea, anagrelide, busulfan, interferon, ruxolitinib, etc.).
